# Supplementary material for: A Meta-Analysis of fMRI Studies of Youth Cannabis Use: Alterations in Executive Control, Social Cognition/Emotion Processing, and Reward Processing in Cannabis Using Youth
Source: Brain Sci. 2022 Sep 23;12(10):1281. doi: 10.3390/brainsci12101281 (PMC9599849; doi:10.3390/brainsci12101281)
Supplement: Supplementary file 1 [file brainsci-12-01281-s001.zip › brainsci-1857840-supplementary.pdf]

Supplementary Online Content

Hammond CJ*, Allick A*, Park G, Rizwan B, Kim K, Lebo R, Nanavati J, Parvaz M, Ivanov I. A Meta-analysis of fMRI Studies of Youth Cannabis Use: Alterations in Executive Control, Social Cognition/Emotion Processing and Reward Processing in Cannabis Using Youth. *Brain Science, 2022*

Methods S1. Additional methodologic details related to task/paradigm domains and meta-analytic procedure

Table S1. Cognitive Domains & Subdomains of Studies used in fMRI meta-analysis

Table S2. Age, Sex, and Cannabis-related Variables in CU youth for use in Meta-analyses and Meta-regression analyses

Table S3. Controlling for covariates/confounders across studies included in the Meta-analysis

Table S4. Proportion of CU Youth with Psychiatric Diagnoses of CUD, AUD, Tobacco Smoking, Depressive disorders, Anxiety disorders, ADHD, and CD/ASPD

Results S1. Qualitative Analysis of fMRI Studies of Brain Activity Differences Between CU vs. TD Youth

Figure S1. Meta-regression Results showing an association between BOLD response differences between CU and TD youth and proportion of female participants

Table S5. Results of Jack-knife Reliability Analyses for Executive Function/Cognitive Control Meta-analysis

Table S6. Results of Jack-knife Reliability Analysis for Social Cognition/Emotion Processing Meta-analysis

Figure S2. Funnel Plots for Primary Meta-analysis for Executive Function/Cognitive Control and Social Cognition Emotion Processing Domains

Table S7. Abstinence-focused Subgroup Meta-analysis of fMRI studies comparing CU and TD youth

Figure S3. Meta-analysis Results showing BOLD response differences in CU compared to TD youth for different abstinence subgroups

Figure S4. Meta-analysis Results showing BOLD response differences in adolescents with CUD compared to TD adolescents

---

Methods S1. Additional methodologic details related to the meta-analytic procedure

<u>Cognitive Domains and Subdomains of Tasks/Paradigms for Subgroup Meta-analysis</u>: We divided studies/experiments into 11 categories based upon common cognitive/affective domains: attention/concentration (ATTN), auditory processing (AUDITORY), decision making (DM), drug cue exposure (DRUG CUE), executive function/cognitive control (EF/CC), interoceptive processes (INT), associative drug memory (MEM), reward processing (REW), self-referential processing (SELF), social cognition/emotion processing (SC/EM), and visuospatial/perceptual-motor (VS/PM). The EF/CC tasks were designed to examine a range of effortful, attention demanding higher order executive

functions including working memory (9 studies/experiments), conflict monitoring (2 studies/experiments), response inhibition (4 studies/experiments), and verbal learning/memory (1 experiment). All of the decision-making tasks measured decision making under risky conditions (5 studies/experiments) and used either gambling paradigms or choice paradigms in the setting of variable risk of monetary loss or gain. The SC/EM tasks all measured brain activation during presentation of social stimuli or emotionally salient stimuli. The social cognition tasks included assessments of decision making, choice behaviors, and BOLD response following social influence and social exclusion. The emotion tasks included emotional perception of facial emotions and affective pictures, cognitive reappraisal of negative emotional stimuli, emotional perception of looming and receding threats, and a task assessing emotional and BOLD response during a Stroop interference task with affective content (affective Stroop task). The REW tasks measured brain activity during different phases of reward processing including reward anticipation and reward receipt/feedback, and in relation to individual differences in the propensity for engaging in reward-related "approach" behaviors using simple win/loss feedback, monetary incentive delay (MID), approach bias, and risky DM tasks.

In our meta-analytic sample of 45 studies: sixteen, five, nine, eight, and six studies were categorized into EF/CC, DM, SC/EM, REW, and DRUG CUE domains respectively. No other domain had more than four studies/experiments. Based upon the number of studies in each category, our primary analyses focused on EF/CC, SC/EM, and REW domains. Focusing on studies for which there were five or more studies/experiments for meta-analytic comparison, supplemental subgroup meta-analyses for DM (5 studies) and DRUG CUE (6 studies) domains and working memory (8 studies), emotion processing (5 studies), and reward feedback (6 studies) subdomains were conducted. Subdomains for which there were too few studies/experiments for an appropriately powered subgroup meta-analysis (e.g., INT with 3 studies), were qualitatively analyzed (see Table S2 and Results S1 below).

<u>Linear meta-regression analyses</u>: Simple linear meta-regression analyses were carried out weighted by the square root of the sample size to predict SDM effect size values. The main output for each variable indicates the regression slope (i.e. amount of BOLD signal change per unit increase in mean age, proportion of females, duration of CU [mean years]), proportion of CUD diagnoses, and mean CUDIT scores. Significant clusters from the individual meta-regression analyses can be interpreted as regions showing BOLD signal differences between CU and TD youth that varied as a function of that variable (e.g., proportion of females) in the datasets/studies.

Table S1. Cognitive Domains & Subdomains of Studies used in fMRI meta-analysis

Domain	Subdomain	Studies	Results
Executive Function & Cognitive Control (EF/CC)	Working Memory (WM)	Padula et al., 2007	Positive (CU>TD)
		Schweinsburg et al. 2005	Positive (CU>TD in mPFC and CU<TD in IFG and temporal regions)
		Schweinsburg et al. 2008	Positive (CU>TD in parietal cortex and CU<TD in mPFC)
		Schweinsburg et al. 2010 (SWM)	Positive (CU > TD)
		Smith et al. 2010	Positive (CU>TD)
		Jager et al., 2010	Negative
		Jacobsen et al., 2007	Positive (CU >TD)
		Kroon et al., 2021 (N-back w/ drug cue)	Positive (WM effect [2-back vs 1-back] and Flanker-by-WM-effect) (CU<TD)
		Tervo-Clemmens et al. 2018	Positive (CU < TD)
	Verbal Learning/Memory	Schweinsburg et al. 2011 (VPAT)	Positive (CU > TD)
	Response Inhibition (RI)	Tapert et al., 2007 (Go/No-Go)	Positive (CU > TD)
		Behan et al., 2014 (Go/No-Go)	Negative
		Thayer et al., 2015 (Stroop)	Negative
		Hatchard et al., 2014 (Stroop)	Positive (CU>TD)
	Conflict Monitoring (CM)	Abdullaev et al. 2010 (ANT)	Positive (CU>TD)
		Cyr et al., 2019	Positive (CU<TD)
Decision Making (DM)	Decision Making Under Risk	DeBellis et al., 2013 (decision reward uncertainty task)	Positive (for uncertain vs. known risk)
		Claus et al., 2018 (BART)	Positive
		Raymond et al., 2020 (BART)	Positive
		Cousijn et al., 2013 (IGT)	Negative (for advantageous vs. disadvantageous DM contrast)
		Aloi et al., 2020 (passive avoidance task)	Negative
Social Cognition & Emotion Processing (SC/EM)	Social Cognition (SC)	Gilman et al., 2016a (social influence)	Positive (CU>TD in mPFC, STG, parietal cortex for social influence vs. no-infl.)
		Gilman et al., 2016b (social exclusion)	Positive (CU<TD in right insula and vmPFC for exclusion vs. fair)
		Gilman et al., 2016c (social influence)	Positive (CU>TD)
		Blair et al., 2021 (retaliation task)	Negative
	Emotion Processing (EM)		
	EM Reactivity	Heitzeg et al., 2015 (affective word stimuli)	Positive (CU < TD in dlPFC, MTG, STG, cuneus, insula, amygdala for negative words and CU > TD in dlPFC and CU < TD in IPL and amygdala for positive words)
		Leiker et al., 2019 (emotional face stimuli)	Positive (CU< TD in rmPFC and ACC for emotional faces [happy vs. netural])
		Blair et al., 2019 (looming threat)	Positive (CU<TD for looming vs. receding threat in mPFC and fusiform)
	EM Regulation	Zimmerman et al., 2017 (cog reappraisal)	Positive for EM regulation (CU>TD in mPFC, cingulate, amygdala during distancing from negative stimuli) but negative for EM reactivity (CU=TD)
		Aloi et al., 2018 (affective Stroop)	Positive (CU>TD)
Reward Processing (REW)	Reward Anticipation	Jager et al., 2013 (MID [win vs. neutral])	Negative
	Reward Feedback	Jager et al., 2013 (MID [win vs. neutral])	Negative
		Acheson et al., 2015 (win/loss feedback task [win vs. neutral and loss vs. neutral])	Positive (win vs. neutral: CU> TD; loss vs. neutral: CU>TD)

		Aloi et al., 2020 (passive avoidance task [reward vs. punishment feedback])<br>DeBellis et al. 2013 (RF during risk-DM [reward vs. no-reward])<br>Cousijn et al., 2013 (RF during risk-DM [win vs. loss])<br>Aloi et al., 2019 (MID task [win vs. loss])	Negative (no main or intxn effects of CUDIT during win vs. loss feedback)<br>Positive (win vs. neutral: CU > TD)<br>Positive (win vs.loss: CU > TD)<br>Positive (CUDIT-by-Accuracy Effect across conditions and CUDIT-by-Reinforcement Cue-by-Accuracy Effect specific to loss feedback/inaccurate trials) [i.e., loss feedback: CU<TD as a function of trial accuracy]; Negative for main effect of CUDIT on BOLD during win feedback, accurate trials.
	Reward-related "Approach" Bias	Aloi et al., 2021b (novelty task)<br>Cousijn et al., 2012 (approach bias SRC w/ drug stimuli [cannabis vs. neutral])	Positive (CU<TD as a function of higher novelty propensity during explore trials)<br>Negative
Associative Memory (MEM)	Implicit Drug Associations	Ames et al., 2013 (MJ implicit assn task)	Positive
Attention (ATTN)	Alerting & Orienting	Abdullaev et al., 2010 (ANT)	Negative for both Alerting & Orienting Contrasts
Visuospatial & Perceptual Motor (VS/PM)	Motor	Lopez-Larson et al., 2012 (motor task)	Positive
Auditory Perception (AUDITORY)	Passive Music Listening	Ford et al., 2014 [MJ-only]<br>Ford et al., 2013 [MJ+MDD]	Negative<br>Positive
Interoceptive Processes (INT)		Migliorini et al., 2013 (soft touch task)<br>Berk et al., 2015 (aversive inspiratory breathing load task)<br>May et al., 2020 (aversive inspiratory breathing load task w/ drug cue)	Positive<br>Positive<br>Positive (for interoceptive contrast)
Self-Referential Processes (SELF)		Aloi et al., 2021 (comparative optimism)	Positive
Drug Cue Exposure	Visual Drug Cue Reactivity	Cousijn et al., 2012a (visual cue)<br>Zhou et al., 2019 [DEP-MJ] (visual cue)<br>Zhou et al., 2019 [ND-MJ] (visual cue)	Negative<br>Positive<br>Positive
	Drug cue exposure during a complex cognitive task (INT, WM, REW tasks)	May et al., 2020 (aversive inspiratory breathing load task w/ visual drug cue)<br>Kroon et al., 2021 (N-back task with cannabis + neutral flanker images)<br>Cousijn et al. 2012b (approach bias SRC task with cannabis + neutral images)	Negative (for drug cue contrast administered as part of INT task)<br>Positive for Flanker-by-WM-effect [CU<TD during cannabis images] but negative for cannabis vs. neutral flanker effect [CU=TD]<br>Negative [CU=TD], but in CU group - BOLD response was correlated with lifetime CU and ΔCUDIT

Notes: Domains: AUDITORY = Auditory Perception; DM = Decision Making (DM); EF/CC = Executive Function/Cognitive Control; L/MEM = Learning & Memory; LAN = Language; ATTN = Complex Attention/Concentration; VS/PM = Visuospatial/Perceptual-motor; SC/EM = Social Cognition/Emotion; REW = Reward; INT = Interoceptive Stimulus Response; Sensory Stimulus Response. Subdomains: DMUR = Decision Making Under Risk; WM = working memory; SOC = Social Cognition; EM = Emotion Processing; EM-Reactivity = Emotion reactivity; EM-regulation = Emotion regulation; RI = Response Inhibition; RS = Response Selection (e.g. choice RT task); CM = Conflict Monitoring; Risky DM = Risk Decision Making; REW-F = Reward Feedback/Receipt; REW-A = Reward Anticipation; DRUG-CUE = Drug Cue-reactivity; MOTOR = motor (e.g. finger tapping task); INT = interoceptive processing; SELF = self-referential processing

Table S2. Age, Sex, and Cannabis-related Variables in CU youth for use in Subgroup Meta-analyses and Meta-regression analyses

Studies	Mean Age CU youth (years)	Proportion of Female participants in CU youth	Proportion of CUD diagnosis in CU youth	Mean CUDIT score for CU youth	Duration of CU (years)	Current CU Frequency[A] (days/episodes per month, avg. past 3 mo.)	Lifetime CU[A] (estimated days/episodes)	Abstinence at MRI scan session	Mean days since last use for CU youth
Padula et al.	18.1	0.18					477.1	≥ 28 days	
Schweinsburg et al. 2005	16.9	0.33	1		3.37	12.8	309.9	≥ 48 hours	
Schweinsburg et al. 2008	18.1	0.27			4	13.5	480.7	≥ 28 days	60.4
Schweinsburg et al. 2011 (verbal paired association test)	18	0.25	0.66		3.2	11	497.8	≥ 21 days	
Schweinsburg et al. 2010 (SWM task)	17.33	0.31			2.5	15.5	428.8	Recent Users (RU): ≥ 24 hours; Abstinent Users (AU): ≥ 27 days	RU: 3.3 days; AU: 38.1 days
Smith et al.	20	0.4			4.55			Ad-lib use	
Tapert et al.	18.1	0.25			4.1	12	475.6	≥ 28 days	58.4
Berk et al.	16.6	0.33	0.73				351.9	≥ 72 hours	
Gilman et al., 2016a	20.6	0.55			2.3	10.8	322.9	≥ 12 hours	
Gilman et al. 2016b	21.4	0.5			6.34	15.6	1305.5	≥ 12 hours	
Gilman et al. 2016c	20.6	0.55	0.5		4.3	11.2	626.1	≥ 12 hours	
Heitzeg et al.	19.8	0.4	0.2		6.4	9.3	618.1	≥ 48 hours	
Migliorini et al.	16.5	0.33	0.73				338.9	≥ 72 hours	
Abdullaev et al.	19.5	0.29			5.1	11	673	≥48 hours	
Cyr et al.	18.9	0.39	1		3.6	20.7	971.4	≥12 hours	3.71
Zhou et al. 2019 (Dependent CU group)	22.9	0	1		5.15			≥ 24 hours	1.66
Zhou et al. 2019	21.5	0	0		4.62			≥ 24 hours	3.47

(Non-Dependent CU group)									
Lopez-Larson et al.	18	0.08			2.9	40	1501	≥12 hours	
Behan et al.	16.5	0.06	1		6.5			≥12 hours	
Acheson et al.	17.6	0.24				26.8		≥12 hours	
DeBellis et al.	16.4	0	1					≥30 days	134
Claus et al.	16	0.28				16.1		≥24 hours	
Jager et al. 2010	17.2	0			4		2003	≥24 hours	35.7
Leiker et al.	16	0.37	0.28	5.48				≥ 30 days	
Blair et al., 2019 (LT)	16.4	0.51	0.32	6.39				≥ 30 days	
Aloi et al., 2021b (novelty task)	16.7	0.39	0.52	9.1				≥ 30 days	
Aloi et al., 2018 (affective stroop)	16.1	0.38	0.35	7.0				≥ 30 days	
Aloi et al., 2019 (MID task)	16.1	0.39	0.37	7.31				≥ 30 days	
Aloi et al., 2020 (PAT)	16.1	0.36	0.44	8.47				≥ 30 days	
Blair et al., 2021 (RT)	16.5	0.34	0.5	9.26				≥ 30 days	
Zimmerman et al., 2017	21.2	0	0.13		4.28	23	1233	≥48 hours	3.58
Ford et al. 2014 (focus on MJ + MDD group)	20.1	0.31				21.3			
Ames et al.	21.1	0.18					500	≥24 hours	
Jacobsen et al. 2007	17.3	0.15			3.7	44.8	847.1	≥30 days	
Jager et al., 2013	17.2	0			4		2003	≥24 hours	35.7
May et al. 2020a	16.6	0.28	0.41				231.5	≥72 hours	45.2
Kroon et al.	21	0.74			5.64	19.52	862.8	≥24 hours	1.28
Hatchard et al., 2014	20	0.4			4.55	45.92		Ad-lib use	
Raymond et al., 2020	21.2	0.53		13.4	4.7	20.8	1270.9	≥12 hours	1.3

Thayer et al., 2015	16	0.26			4.49	4.5	243		
Aloi et al., 2021a (COT)	16.3	0.43	0.59	10.3				≥30 days	
Tervo-Clemmens et al., 2018	15.6	0.45			1.91			≥24 hours	
Cousijn et al., 2012a (drug cue reactivity)	21.4	0.35	0.52	12.6	2.5	20	650	≥24 hours	
Cousijn et al., 2012b (Approach bias)	21.3	0.36		12.4	2.5	19.6	637	≥24 hours	
Cousijn et al., 2013 (IGT)	21.4	0.34		12.2	2.5	16	520	≥24 hours	

Note: Unfilled/blank boxes in the table represent studies where the variable of interest was not reported/provided or could not be calculated from other items. [A]There was a large amount variability in how current and lifetime cannabis use frequency was assessed and reported across studies (e.g., different reported cannabis outcomes: number of joints vs. grams vs. use episodes vs. occasions vs. days used; different reported time windows: past week, past-28-days, past-30-days, past-month, past-3-months, past-year, lifetime). For our meta-regression analyses, we attempted to create harmonized current and lifetime cannabis use frequency variable across studies focusing on days/occasions of use in the past 30 days, averaged over the past-3-months (for current use) and days/occasions of lifetime use (for lifetime use) but these estimates were unreliable. Given this, we elected to not conduct formal meta-regression analyses using these variables instead choosing to conduct meta-regression analyses on two variables that we thought were more reliable – CUD diagnoses, CUDIT scores, and duration of CU. CUD diagnoses made by clinician or by research staff using semi-structured interviews. CUDIT is a validated measure of CUD severity. Duration of CU does have more variability than CUD and CUDIT but was retained because it is easier to recall for informants and to approximate compared to lifetime use episodes. Still, it is important to note that this variable did have some cross-study variation as some studies reported duration of regular use and others reported duration of use and other studies reported on age of cannabis initiation or age of onset of regular use and this was subtracted from these youth's current age to calculate a duration of use variable. Given the high degree of variability in outcomes and reporting - the field should work toward developing and using a common outcome set (COS) of validated measures and outcome variables for use in future studies.

Table S3. Controlling for covariates/confounders across studies included in the Meta-analysis

**Studies**	Attempted to Control for alcohol use	Attempted to Control for tobacco use	Excluded youth with comorbid psychiatric disorders	Excluded youth with psychotropic medication use
Padula et al. 2007	No	No	Yes (excluded adolescents with psychiatric comorbidities, ADHD, conduct disorder, and substance use disorders other than alcohol or cannabis)	Yes
Schweinsburg et al. 2005	Yes	No	Yes (excluded adolescents with psychiatric comorbidities, ADHD, substance use disorders	Not reported

			other than alcohol or cannabis and conduct disorder)	
Schweinssburg et al. 2008	Yes	No	Yes (excluded adolescents with psychiatric comorbidities, ADHD, substance use disorders other than alcohol or cannabis, tobacco use disorder)	Not reported
Schweinsburg et al. 2010 (SWM)	Yes, examined relationship between BOLD response and potentially confounding factors (other SU and CD)	No	Yes (excluded adolescents with history of Axis I psychiatric comorbidities, ADHD, substance use disorders other than alcohol or cannabis, tobacco use disorder)	Not reported
Smith et al.	Yes	Yes, controlled for nicotine use	Yes, screened out youth with Axis I psychiatric disorders or tested positive for cocaine, opiates, amphetamines	Not reported
Tapert et al. 2007	No	Yes, completed Fagerstorm test for Nicotine Dependence	Yes (excluded adolescents with psychiatric comorbidities, ADHD, conduct disorder, substance use disorders other than alcohol or cannabis, tobacco use disorder)	Yes
Berk et al. 2015	No	Yes, partially. The authors ran supplemental analyses examining cigarette use as predictor of BOLD response	Yes, screened out youth with Axis I psychiatric disorders or substance use disorders other than alcohol or cannabis	Yes
Gilman et al. 2016a	No	No	Yes, excluded if pt met criteria for Axis I and II, except CUD	No participant was taking any medication
Gilman et al. 2016b	No	No	Yes, excluded. All participants were healthy, no current psych diagnosis	NP
Gilman et al. 2016c	No	No	Yes, excluded those who meet criteria for DSM-4 Axis I disorder	NP

Heitzeg et al.	Yes, identified controls with similar alcohol/nicotine use	Yes, identified controls with similar alcohol/nicotine use	Presence of active primary Axis I disorder were exclusion, however untreated mood, anxiety, APD and SUD were included	Yes
Migliorini et al.	Yes	No	Excludes pts with presence of any DSM-IV axis I psychiatric disorder	Yes
Abdullaev et al.	Yes, excluded from control and MJ group if they used alcohol >1-2 day/week	"also exclused if they reported using other drugs", doesn't specify nicotine	NP	NP
Cyr et al.	Yes	No	Did not exclude DSM-5	NP
Zhou et al. 2019 Dependent MJ Group	Controlled for alcohol use	Controlled for nicotine use	Exclusion of DSM-IV Axis I and II, DBI >20, current medical disorder	Yes, excluded pts currently taking medication
Zhou et al. 2019 Non-Dependent MJ group	Controlled for alcohol use	Controlled for nicotine use	Exclusion of DSM-IV Axis I and II, DBI >20, current medical disorder	Yes, excluded pts currently taking medication
Lopez-Larson et al.	Yes, excluded pt with alcohol dependence (2 months prior to scan)	No	Health controls – excluded DSM-IV axis I dx, does not mention MJ group having similar exclusion criteria, with exception of exclusion for autism, schizophrenia, anorexia, and drug/alcohol dependence	No
Behan et al.	Yes, controlled for alcohol use	Yes, controlled for nicotine use	Participants were screened for no history of neurological/psychiatric illness or any past loss of consciousness which required hospitalization.	NP
Acheson et al.	Yes, controlled for alcohol use	Yes, controlled for nicotine use	Exclusionary criteria included physical or neurological conditions that would interfere with task performance, DSMI V Axis I psychiatric disorder (other than cannabis use disorders)	NP
DeBellis et al.	Yes, controlled for alcohol use	Yes, controlled for nicotine use	Exclusion criteria for subjects were medical,	No subjects were taking psychotropic medication

			pervasive developmental or psychotic disorder	
Claus et al.	Yes, controlled for alcohol use	Yes, reported nicotine use last month	NP	NP
Jager et al. 2010	Yes, controlled for alcohol use	Yes, controlled for nicotine use	Excluded Axis I except for conduct disorder	Yes, use of psychotropic medication was an exclusion criteria
Leiker et al. 2019	Yes, controlled for alcohol use	Yes, controlled for nicotine use	Exclusion criteria included PDD, TS, history of psychosis, neurological disorders, head trauma	Yes, excluded patients taking medication with psychotropic effects
Blair et al., 2019 (Looming Threat task)	Yes, controlled for alcohol use	Yes, controlled for nicotine use	Current psychiatric conditions (other than psychotic disorders or pervasive developmental disorders) were not exclusionary.	Use of psychotropic medications for psychiatric indications (e.g., stimulants, selective serotonin reuptake inhibitors) were not exclusory. However, participants on stimulant medication were asked to withhold medication on the day of scanning.
Aloi et al., 2021b (novelty task)	Did not control for alcohol use	Did not control for nicotine use	Current psychiatric conditions (other than psychotic disorders or pervasive developmental disorders) were not exclusionary.	Use of psychotropic medications for psychiatric indications (e.g., stimulants, selective serotonin reuptake inhibitors) were not exclusory. However, participants on stimulant medication were asked to withhold medication on the day of scanning.
Aloi et al. 2018 (affective stroop task)	Yes, controlled for alcohol use	NP	Exclusion criteria included pervasive developmental disorder, Tourette's syndrome, lifetime history of psychosis, neurological disorder, head trauma, and non-psychiatric medical illnesses requiring medications	Excluded non-psychiatric medical illnesses requiring medications that may have psychotropic effects

			that may have psychotropic effects	
Aloi et al. 2019 (MID task)	Yes, controlled for alcohol use	Yes, controlled for nicotine use	Current psychiatric conditions (other than psychotic disorders or pervasive developmental disorders) were not exclusionary	Current psychotropic use were not exclusionary, except asked to hold stimulant for those on stimulants
Aloi et al., 2020 (passive avoidance task)			Current psychiatric conditions (other than psychotic disorders or pervasive developmental disorders) were not exclusionary	Current psychotropic use were not exclusionary, except asked to hold stimulant for those on stimulants
Blair et al., 2021 (retaliation task)	Yes, controlled for alcohol use	NP	Did not exclude those with psychiatric disorder	NP
Zimmerman et al., 2017	Yes, controlled for alcohol use	Yes, controlled for nicotine use	Exclusion criteria for all participants history of psychiatric disorder according to DSM-IV criteria (assessed using the Mini-International Neuropsychiatric Interview (M.I.N.I.), Sheehan et al. [1998])	Excluded regular or current use of psychoactive
Ford et al. 2014 MJ Group	Yes, controlled for alcohol use	Yes, controlled for tobacco use	No participants in the MJ group met criteria for a current or past depressive episode	NP
MDD + MJ Group	Yes, controlled for alcohol use	Yes, controlled for tobacco use	Participants met criteria for MDD + MJ	The participants included in the MDD group met current criteria for a major depressive episode, while those in the MDD + MJ groups met diagnostic criteria for either current or past MDD, and a total of 13 participants were taking psychoactive medications (primarily SSRIs).
Ames et al. 2013	No	No	Participants excluded if they have a psychiatric disorder	Excluded if using psychotropic medication
Jacobsen et al. 2007	Yes, controlled for alcohol use	Yes, controlled for nicotine use	All participants were recruited from the community and were free of medical and	All participants were recruited from the community and were free of medical and

			psychiatric illness and substance abuse or dependence disorders	psychiatric illness and substance abuse or dependence disorders
Schweinsburg et al. 2011 (verbal paired word association test)	Yes, controlled for alcohol use	No participants were regular smokers	Exclusions were teen history of medical or neurological disorders, DSM-IV psychiatric diagnoses other than alcohol or marijuana use disorder	NP
Jager et al., 2013	Yes, controlled for alcohol use	Yes, controlled for nicotine use	Axis I psychiatric diagnosis, except for conduct disorder (which is a common diagnosis in cannabis using boys)	Excluded use of psychotropic medication
May et al. 2020	Yes, controlled for alcohol	Yes, controlled for nicotine	Participants were excluded if they endorsed any of the following: (1) lifetime Diagnostic and Statistical Manual (DSM-5) of Mental Disorders psychiatric disorder (other than substance use disorder, SUD)	Excluded patients with current use of psychoactive medication
Kroon et al. 2021	Yes, controlled for alcohol use	Yes, controlled for nicotine use	Exclusion criteria mental health (major axis-1 disorders) problems, and previous or current treatment for CUD or plans to enter treatment.	Exclusion criteria current use of prescription or illicit psychoactive drugs
Hatchard et al. 2014	Yes, controlled for alcohol use	Yes, controlled for nicotine use	Excluded participants that met diagnostic criteria for an Axis I diagnosis from the Diagnostic and Statistical Manual of Mental Disorders (DSM)	NP
Raymond et al. 2020	Yes, controlled for alcohol use	NP	All participants had to be free of psychological disorders (with the exception of cannabis use disorder for the CB group)	NP
Thayer et al. 2015	Yes, controlled for alcohol use	NP	Participants were not specifically excluded on the basis of any psychiatric disorders	But excluded rather only if they indicated psychotropic medication use suggestive of

			including Attention Deficit Hyperactivity Disorder (ADHD)	greater severity of psychopathology.
Aloi et al. 2021a (CO Task)	Yes, controlled for alcohol use	NP	Current psychiatric conditions (other than psychotic disorders or PDD) were not exclusionary	. Use of psychotropic medications for psychiatric indications (e.g., stimulants, selective serotonin reuptake inhibitors) were not exclusory.
Tervo-Clemmens et al. 2018	Yes, controlled for alcohol use	NP	Excluded current psychiatric disorder	Excluded current psychotropic medication use
Cousijn et al. 2012a	Yes	Yes	Excluded major medical disorders or a history of major type I psychiatric disorders, which was assessed with the Mini-International Neuropsychiatric Interview	NP
Cousijn et al. 2012b	Yes, controlled for alcohol use	No, could not control for nicotine use	Excluded major medical disorders or a history of major type I psychiatric disorders, which was assessed with the Mini-International Neuropsychiatric Interview	NP
Cousijin et al. 2013	Yes	Yes	Excluded major medical disorders or a history of major type I psychiatric disorders, which was assessed with the Mini-International Neuropsychiatric Interview	NP

Abbreviation: NP = not provided

Table S4. Proportion of CU Youth with Psychiatric Diagnoses of CUD, AUD, Tobacco Smoking, Depressive disorders, Anxiety disorders, ADHD, and CD/ASPD[a]

Studies	Diagnostic Assessments[b]	Proportion of CUD	Proportion of AUD	Proportion of tobacco smokers	Proportion of depressive disorders or MDD	Proportion of anxiety disorders or GAD	Proportion of ADHD (across subtypes)	Proportion of conduct disorders/ASPD
Padula et al. 2007	Computerized DISC-PS-4.32	NP	0.12 (2/17)	NP	0.00 (0/17)	0.00 (0/17)	0.00 (0/17)	0.00 (0/17)
Schweinsburg et al. 2005	CDDR and DISC	1.0 (15/15)	1.0 (15/15)	0.46	0.00 (0/15)	0.00 (0/15)	0.00 (0/15)	0.27
Schweinsburg et al. 2008	Computerized DISC-PS-4.32	NP	0.27 (4/15)	0.27 (4/15) (past month tobacco use)	0.00 (0/15)	0.00 (0/15)	0.00 (0/15)	0.00 (0/15)
Schweinsburg et al., 2011 (VPAT)	DISC	0.66 (average from MJ only (n=8) = 0.50 and MJ+BD (n=28) = 0.71)	0.28 (average from MJ only (n=8) = 0.0 and MJ+BD (n=28) = 0.36)	NP	0.00	0.00	0.00	0.00
Schweinsburg et al. 2010 (SWM)	DISC	NP	.10 (estimated as 2.5/26 from 5 of 26 MJ patients having AUD or CD)	NP	0.0	0.0	0.0	.10 (estimated as 2.5/26 from 5 of 26 MJ patients having AUD or CD)
Smith et al. 2010	Computerized DISC	NP	0.0	0.7 (7/10 current smokers)	0.00 (0/10)	0.00 (0/10)	0.00 (0/10)	0.00 (0/10)
Tapert et al. 2007	DISC-PS-4.23 CDDR	NP	0.0	NP	0.00 (0/16)	0.00 (0/16)	0.00 (0/16)	0.00 (0/16)
Berk et al. 2015	SSADDA and clinical assessment by psychiatrist and psychologist	0.73 (11/15)	0.27 (4/15)	NP	0.00 (0/15)	0.00 (0/15)	0.00 (0/15)	0.00 (0/15)
Gilman et al. 2016a (Social Influence Task)	SCID		0.00 (0/20)	0.35 (7/20 occasional smokers)	0.00 (0/20)	0.00 (0/20)	0.00 (0/20)	0.00 (0/20)
Gilman et al. 2016b (Social Influence DM task)	SCID		0.00 (0/20)	0.40 (8/20 occasional smokers; 1/20 daily smoker)	0.00 (0/20)	0.00 (0/20)	0.00 (0/20)	0.00 (0/20)
Gilman et al. 2016c (Cyberball Social Exclusion task)	SCID	0.50 (8/20) Cannabis abuse – 0.40 Cannabis dependence – 0.20	0.00 (0/20)	0.00 (0/20 regular smokers)	0.00 (0/20)	0.00 (0/20)	0.00 (0/20)	0.00 (0/20)

Heitzeg et al. 2015	DISC <18; DIS-Version IV for >18 yo	0.20 (4/20)	0.35 (7/20)	0.35 (7/20 current smokers)	0.30 (6/20)	0.30 (6/20)	NP	0.35 (7/20) *APD dx
Migliorini et al. 2013	SSADDA and DISC	0.73 (11/15)	0.27 (4/15)	NP	0.0	0.0	0.0	0.0
Abdullaev et al. 2010	NP	NP	NP	NP	NP	NP	NP	NP
Cyr et al. 2019	SCID-I and K-SADS-PL	1.0 (Cannabis abuse 0.43 Cannabis dependence 0.61)	0.04 (1/28)	NP	0.07 (2/28)	0.04 (1/28)	0.17 (4/28)	NP
Zhou et al. 2019-DEP	MINI for DSM-IV	1.00 (100% lifetime cannabis dependence) (18/18)	NP	0.94 (17/18 tobacco smokers)	0.00	0.00	0.00	0.00
Zhou et al. 2019-ND	MINI for DSM-IV	0.0 (20/20)	NP	.70 (14/20 tobacco smokers)	0.00	0.00	0.00	0.00
Lopez-Larson et al. 2012	SCIDS-P, K-SADS-PL, Diagnostic semi structured interview by board certified child psychiatrist or licensed psychologist	NP	0.17 (4/24 current alcohol abuse)	0.33 (4/24 current tobacco users)	0.00	0.00	0.00	0.00
Behan et al. 2014	WHO CIDI-SF	1.0 (17/17)	NP	NP	0.00	0.00	0.00	0.00
Acheson et al. 2015	SCID for DSM-IV and assessment of psychiatric health and drug/alcohol use history	NP	0.0 (0/14 AUD diagnosis; 4/14 drank alcohol weekly)	0.36 (5/14 current tobacco use)	0.00	0.00	0.00	0.00
DeBellis et al. 2013	KSADS-PL	1.0 (15/15)	0.26 (4/15)	0.26 (4/15)	0.60 (9/15)	0.73 (11/15)	0.47 (7/15)	0.53 (8/15)
Claus et al. 2018	Risky Behavior questionnaire and Time Line Follow-Back	NP	NP	0.67 (avg. across MJ and MJ+ALC groups)	NP	NP	NP	NP
Jager et al. 2010	C-DISC	NP	NP	NP	0.00 (0/12)	0.00 (0/12)	0.00 (0/12)	0.75 (9/12)

Leiker et al. 2019	Clinical interviews by licensed psychiatrist + CUDIT AUDIT	0.28 (29/104) CUDIT>8	0.23 (7/104) AUDIT>4	NP	0.17	0.25	0.49	0.38
Blair et al., 2019 (Looming Threat task)	Clinical interviews by licensed psychiatrist + CUDIT AUDIT	0.32 (28/87) CUDIT>8	0.24 (21/87) AUDIT>4	NP	0.22	0.39	0.52	0.53
Aloi et al., 2021b (novelty task)	Clinical interviews by licensed psychiatrist + CUDIT AUDIT	0.52 (67/128) CUDIT>6	0.31 (40/128) AUDIT>4	NP	0.17	0.34	0.50	0.48
Aloi et al. 2018 (affective stroop task)	Clinical interviews by licensed psychiatrist + CUDIT AUDIT	0.35 (29/82) CUDIT>8	0.26 (21/82) AUDIT>4	NP	0.26	0.30	0.52	0.52
Aloi et al. 2019 (MID task)	Clinical interviews by licensed psychiatrist + CUDIT AUDIT	0.37 (56/150) CUDIT>8	0.31 (38/150) AUDIT>4	NP	0.29	0.24	0.53	0.51
Aloi et al., 2020 (passive avoidance task)	Clinical interviews by licensed psychiatrist + CUDIT AUDIT	0.44 (62/141) CUDIT>8	0.31 (43/141) ADUIT>4	NP	0.15	0.28	0.53	0.47
Blair et al., 2021 (retaliation task)	Clinical interviews by licensed psychiatrist + CUDIT AUDIT	0.50 (51/102) CUDIT>8	0.29 (30/102) AUDIT>4	NP	0.14	0.31	0.51	0.49
Zimmerman et al., 2017	MINI	0.13 (3/23)	0.0	0.74 (17/23 smokers)	0.00	0.00	0.00	0.00
Ford et al. 2014 MDD+ MJ Group	Clinical interview by licensed psychiatrist confirmed by SCID for DSM-IV	NP	NP	NP	1.00	NP	NP	NP
Ames et al. 2013	Patient self-reported history of psychiatric or neurologic disorders and heavy drinking done via screening questions	NP	NP	NP	NP	NP	NP	NP
Jacobsen et al. 2007	SCID	NP	NP	1.00 (20/20)	0.00 (0/20)	0.00 (0/20)	0.00 (0/20)	0.00 (0/20)
Jager et al., 2013	C-DISC	NP	NP	NP	0.00	0.00	0.00	0.43 (9/21)

May et al. 2020	Clinical interview SSADDA	0.41 Avg. across groups - CAN+ALC-SUD group: 0.92; CAN+ALC-EXP group: 0.00	0.61	NP	0.00	0.00	0.00	0.00
Kroon et al. 2021	Pt self-reported psychiatric history done via telephone screening	NP	0.0 0 with AUDIT > 12	0.47	NP	NP	NP	NP
Hatchard et al. 2014	Parent and Patient Assessments conducted as part of Ottowa Prenatal Prospective Study; no details on specific questions or diagnostic assessments	NP	NP	0.7 (7/10 cigarette smokers)	0.00	0.00	0.00	0.00
Raymond et al. 2020	SCID	NP	NP	NP	0.00	0.00	0.00	0.00
Thayer et al. 2015	Substance use history through self-report	NP	NP	NP	NP	NP	NP	NP
Aloi et al. 2021a (CO Task)	Clinical interviews by licensed psychiatrist + CUDIT AUDIT	0.59 (61/104) CUDIT>8	0.43 (45/104) AUDIT>4	0.15 (16/104)	0.26	0.27	0.46	0.50
Tervo-Clemmens et al. 2018	MINI	NP	0.0	0.05 (1/22 cigarette user)	0.00	0.00	0.00	0.00
Cousijn et al. 2012a	MINI for DSM-IV, CUDIT, AUDIT	.52 (16/31 cannabis abuse or dependence)	0.0 AUDIT > 8 exclusionary	0.68 (cigarette smoker status)	0.00	0.00	0.00	0.00

Cousijn et al. 2012b	MINI for DSM-IV, CUDIT, AUDIT	NP	0.0<br>AUDIT > 8 exclusionary	0.70<br>(cigarette smoker status)	0.00	0.00	0.00	0.00
Cousijin et al. 2013	MINI for DSM-IV, CUDIT, AUDIT	NP	0.0<br>AUDIT > 8 exclusionary	0.69<br>(cigarette smoker status)	0.00	0.00	0.00	0.00

a= Proportion of CU youth with respective psychiatric diagnoses from each study are presented as a proportion with 0.75 representing 75% of youth from the CU sample. The number of participants with each diagnosis and the total number of CU Youth in each study is shown in ( ) below the proportions (e.g. no. participants with X diagnosis / no. CU youth participants). b= Diagnostic Assessment used to obtain the psychiatric diagnoses for participants in each study. Abbreviations: ADHD = Attention Deficit/Hyperactivity Disorder; ASPD = Antisocial Personality Disorder; AUD = Alcohol Use Disorder; CD = Conduct Disorder; CUD = Cannabis Use Disorder; TUD = Tobacco Use Disorder; CUDIT = Cannabis Use Disorder Identification Test; AUDIT = Alcohol Use Disorder Identification Test; NP = Not provided; DSM = Diagnostic and Statistical Manual of Psychiatric Disorders; Pt = patient; DISC, C-DISC, DISC-PS-4.23 = variations of the Diagnostic Interview Schedule for Children; KSADS, KSADS-PL = versions of the Kiddie Schedule for Affective Disorders and Schizophrenia; MINI = Mini International Neuropsychiatric Interview; SCID = Structured Clinical Interview for DSM Psychiatric Disorders; SSADDA = Semi-Structured Assessment for Drug Dependence and Alcoholism; WHO CIDI-SF = World Health Organization Composite International Diagnostic Interview, short form.

Results S1. Qualitative Analysis of Studies of BOLD signal Differences Between Cannabis Using and Non-Using Typically Developing Youth

<u>Qualitative summary</u>: Forty-five studies/experiments using whole-brain voxel-wise analyses to compare BOLD signal differences between CU and TD youth met all inclusion criteria and were included in the qualitative and quantitative analyses. Out of the 45 fMRI studies, 37 (82%) reported either differences between CU and TD youth or brain-behavior associations with CU variables in combined samples. Thirty-six of the 45 fMRI studies reported group-level comparisons between CU and matched non-using TD youth and nine fMRI studies reported results from correlational analyses between brain activation measures (i.e. BOLD signal) and cannabis use outcomes in combined samples of CU and TD youth.

<u>Heterogeneity vs. Homogeneity of Experimental Design, Analytic Approaches, and Sample Characteristics of Studies</u>: There was heterogeneity across study designs, analytic methods used, and sample characteristics for the 45 studies. Many studies had small samples and were underpowered. A number of studies used region-of-interest (ROI) analyses and small volume corrections (SVCs) in their primary analyses and conducted exploratory whole-brain analyses. For these studies, only results from the exploratory whole-brain analyses were included in the quantitative analysis. A majority of the studies attempted to control for potentially confounding variables either through restricting the study sample, controlling for covariates in the statistical analyses, or taking both of these steps (see eTable S3). Regarding sampling procedures, there was wide variability in inclusion/exclusion (I/E) criteria for co-occurring alcohol and tobacco/cigarette use and comorbid psychiatric disorders across the studies. Generally studies fell into one of two camps with some authors applying strict I/E criteria (i.e., excluding youth with psychiatric disorders) with the goal of having a "clean" CU sample free of comorbid psychiatric conditions that could confound fMRI results and other authors applying more lenient I/E criteria (i.e., including youth with comorbid/co-occurring psychiatric disorders) with the goal of having a generalizable "real-world" CU sample and then controlling for variance related to comorbidity at the analysis stage. The proportion of CU youth with co-occurring/comorbid alcohol use disorder (AUD), regular tobacco/cigarette use, depressive disorders, anxiety disorders, attention deficit/hyperactivity disorder (ADHD), and conduct disorder (CD) or antisocial personality disorder (ASPD) from studies that used standardized or clinical interviews is shown in eTable S4.

<u>*Whole-brain Voxel-wise Group-level Comparisons of BOLD effect between CU vs. TD youth*</u>: Focusing on the group-level comparisons (n=36 studies): thirty-one studies (86% of the group-comparison subset sample) showed BOLD signal differences between CU and non-using TD youth in whole-brain voxel-wise analyses.

<u>*Whole-brain Correlation Analyses in Combined Samples of CU and TD youth*</u>: Of the nine studies that investigated relationships between BOLD fMRI signal and cannabis outcomes as their main *a priori* analysis, six (67%) reported significant correlations between cannabis-related variables and brain activation.

<u>*Spatial Distribution of Adolescent Cannabis Use Effects and Evidence for Distinct Regional Patterns of Brain Activity Related to Cannabis Use During Adolescence*</u>: No global BOLD signal differences were observed. FMRI studies that used whole-brain voxel-wise analyses and showed significant BOLD signal differences between CU and TD youth or brain-behavior correlations reported regionally specific effects which were broadly distributed across prefrontal, temporal, and parietal networks in some studies and narrow/focal to a specific region in others. Cortical and subcortical brain regions that showed BOLD response differences between CU and TD youth across studies included the amygdala, hippocampus, parahippocampal gyrus, nucleus accumbens (NAc), ventral tegmental area (VTA), putamen, caudate, globus pallidus, ventral striatum (VS), brain stem, thalamus, anterior and posterior insula, uncus, culmen, all subsections of the cingulate (dorsal anterior cingulate cortex (dACC), mid-cingulate, posterior cingulate), precuneus, cuneus, lingual gyrus, angular gyrus, postcentral gyrus, fusiform gyrus, rolandic operculum, precentral gyrus, claustrum, declive, superior parietal lobule, inferior parietal lobule (IPL), superior temporal gyrus (STG), temporal pole, middle temporal gyrus (MTG), inferior temporal gyrus (ITG), supramarginal gyrus (SMG), dorsolateral prefrontal cortex (dlPFC), ventral medial prefrontal cortex (vmPFC), orbitofrontal cortex (oPFC), rostral medial prefrontal cortex (rmPFC), dorsal medial prefrontal cortex (dmPFC), frontal pole, middle frontal gyrus (MFG), superior frontal gyrus (SFG), inferior frontal gyrus, supplementary motor area (SMA), Pre-SMA, occipital lobe, middle occipital gyrus, lateral occipital gyrus, cerebellum, and cerebellar lingual gyrus. Across studies activation differences in CU compared to TD youth were most consistently observed in medial prefrontal, cingulate, insula, and temporal cortical regions as well as in subcortical regions implicated in reward and emotion processing. These regions are notable for having elevated expression of CB1 receptors compared to the rest of the brain.

Does Adolescent Cannabis Use Impact Some Cognitive Domains and Spare Others? Regarding differences in CU versus TD brain activity for distinct cognitive domains: eTable S1 presents the studies that investigated distinct cognitive domains and subdomains and the results from those studies. As noted above: sixteen, five, nine, eight, and six studies were categorized into EF/CC, DM, SC/EM, REW, and DRUG CUE domains respectively, with fewer than four studies/experiments present in all other domains (1in MEM, 1 in ATTN, 1 in VS/PM, 2 in AUDITORY, 3 in INT, and 1 in SELF). Group differences and cannabis-related brain-behavior associations were most consistent in EF/CC and SC/EM domains where thirteen out of sixteen (81% of EF/CC experiments) and eight out of nine (89% of SC/EM experiments) showed significant CU vs. TD differences in brain activation. EF/CC studies with positive results frequently showed differences in prefrontal, temporal, and parietal cortical regions with subcortical differences being less common. Positive primary results were less consistent but still present in over 50% of studies/experiments using DM, REW, and DRUG CUE paradigms. For DM under risky conditions, three out of five studies showed positive results. Five out of eight REW studies showed BOLD differences between CU and TD youth with the majority of these studies using reward feedback contrasts. Studies examining drug cue exposure had inconsistent results (3 of 6 experiments showed positive results) although some of this variance may be related to variability in task/paradigm. Of the domains with fewer than four experiments/studies, notable qualitative findings were observed related to interoceptive processing tasks (3 studies, all positive). In examining EF/CC subdomain results: Differences in brain activation between CU and TD groups were consistently observed during WM tasks (8 of 9 positive) and CM tasks (2 of 2 positive) and to a lesser extent during RI tasks (2 of 4 positive). Results of the SC/EM subdomain qualitative analysis indicate that neural responses during the processing of social information (SC: 3 of 4 positive), negative emotional stimuli (emotion reactivity), and when individuals are required to regulate their behaviors when in the presence of negative emotional stimuli (emotional regulation) (EM: 5 of 5 positive) are altered in CU compared to TD youth. Notably, three of four task contrasts assessing neural response to emotional stimuli (a measure of emotional reactivity) (all 3 showing CU < TD) and two of two contrasts assessing emotion regulation (both CU > TD) showed CU vs. TD differences in brain activity. In examining REW subdomain results: It is important to note that while five of eight REW studies showed CU vs. TD BOLD differences, two of these studies only showed positive results as a function of trial accuracy (Aloi et al., 2019) or self-reported novelty (Aloi et al., 2021b) and showed no CU vs. TD differences with traditional reward contrasts (i.e., no main

effect for win vs. loss feedback). Three of six studies (50%) examining reward feedback contrasts showed CU vs. TD group differences. Among reward feedback studies, two reported on outcomes of win vs. neutral feedback (1 of 2 positive) (Jager et al., 2013; De Bellis et al., 2013), one reported on outcomes of both reward vs. neutral and loss vs. neutral feedback (both positive) (Acheson et al., 2015), and three reported on outcomes of win vs. loss feedback (1 of 3 positive) (Aloi et al., 2019; Aloi et al., 2021b; Cousjin et al., 2013). Only one whole-brain study examined reward anticipation (Jager et al., 2013). The results of this study showed no CU vs. TD differences in anticipatory processing during win vs. neutral trials.

Is Adolescent Cannabis Use Associated with Increased Brain Activity, Decreased Brain Activity, or Neither? In terms of directional relationships across the group-comparisons (n=36): twenty-one studies (58%) reported increased BOLD response, six studies (17%) reported decreased BOLD response, four studies (11%) reported both increased and decreased BOLD response (in different regions and/or for different contrasts), and five studies (14%) reported neither increased nor decreased BOLD response (i.e., null finding) between CU and matched non-using TD youth. For studies examining brain-behavior associations between cannabis variables and BOLD response in combined samples (n=9 studies), one study that used an affective Stroop task to assess emotion regulation reported a positive correlation between CUD severity and BOLD response (i.e. ↑ CUD severity = ↑ brain activity in CU compared to TD youth), five studies (56%) reported a negative correlation between CUD severity and BOLD response (i.e., ↑ CUD severity = ↓ brain activity in CU compared to TD youth), and 3 studies (33%) reported no significant relationship between CU frequency or CUD severity and BOLD response. Of note, the cannabis variable used in the primary analyses of all but one these brain-behavior correlation studies was the mean CUDIT score. Focusing on specific domains across all study types: In examining EF/CC results (n=16 studies): seven studies (44%) reported increased BOLD response, three studies (19%) reported decreased BOLD response, three studies (19%) reported both increased and decreased BOLD response in different regions or during different contrasts, and three studies (19%) reported no differences between CU and TD youth. Focusing on EF/CC subdomains: Working Memory (WM) (8 studies): Four WM studies showed increased BOLD response, two WM studies showed decreased BOLD response, and two WM studies reported regions with increased and decreased BOLD response in CU compared to TD youth. Response Inhibition (RI)(4 studies): Two RI studies showed increased BOLD response and

two RI studies showed no difference in BOLD response between CU and TD youth. Conflict Monitoring (CM) (2 studies): One CM study showed increased BOLD response and the other showed decreased BOLD response in CU compared to TD youth. In examining SC/EM results (9 studies): four experiments reported increased BOLD response, four experiments reported decreased BOLD response, and two experiments reported null findings with regard to CU vs. TD differences. Findings with regard to neural response in the SC domain varied by social cues and task type. For example, CU showed increased activation compared to TD youth in the two SC studies using social influence paradigms (Gilman et al., 2016a, 2016b) but showed decreased activation compared to TD youth in a report that used a social exclusion paradigm (Gilman et al., 2016c) and did not differ from TD youth on a retaliation task (Blair et al., 2021). In the EM subdomain, CU status was associated with decreased brain activity during emotional reactivity (CU < TD) and increased brain activity during emotion regulation (CU > TD). For example, three of four studies measuring neural response during passive viewing of negative affective stimuli (i.e., emotion reactivity) showed decreased BOLD response in CU vs. TD youth. In contrast, two of two studies measuring neural response while youth were attempting to regulate their emotions or behaviors in the presence of negative affective stimuli (i.e., emotion regulation) showed increased BOLD response in CU vs. TD youth. These qualitative results complement our meta-analytic findings for EM. Taken together, they suggest that adolescent cannabis use may result in emotion dysregulation with blunted 'bottom-up' emotional reactivity and increased recruitment of compensatory PFC systems required to maintain behavioral control during emotion regulation. In examining REW results (n=8 studies, 9 experimental contrasts): three experiments reported increased BOLD response, four experiments reported no difference in BOLD response, and two experiments reported decreased BOLD response in CU compared to TD youth. However, as noted above the experiments showing decreased BOLD response in CU compared to TD youth should be interpreted cautiously as they reflect reward related differences in BOLD response among CU and TD youth that were only seen when trial accuracy and self-reported novelty were included in the models, with the main reward contrasts for these studies showing no differences between CU and TD youth. Focusing on traditional reward feedback contrasts: three experiments (50%) reported increased BOLD response (2 win vs. neutral and 1 win vs. loss), no experiments (0%) reported decreased BOLD response, and three experiments (50%) reported no difference in BOLD response (1 win vs. neutral and 2 win vs. loss) between CU and TD youth. One whole-brain study examined reward (win vs. neutral) and punishment (loss vs. neutral) feedback separately (Acheson et

al., 2013). The results of this study showed that CU, when compared to TD controls, had increased BOLD response in left lateralized mPFC regions and the left caudate during reward feedback and in right lateralized mPFC regions along with the right ACC, right posterior cingulate, and left insula during punishment feedback. The only whole-brain study to examine reward anticipation showed no CU vs. TD group differences.

Figure S1. Meta-regression Results showing an association between BOLD response differences between CU and TD youth and proportion of female participants

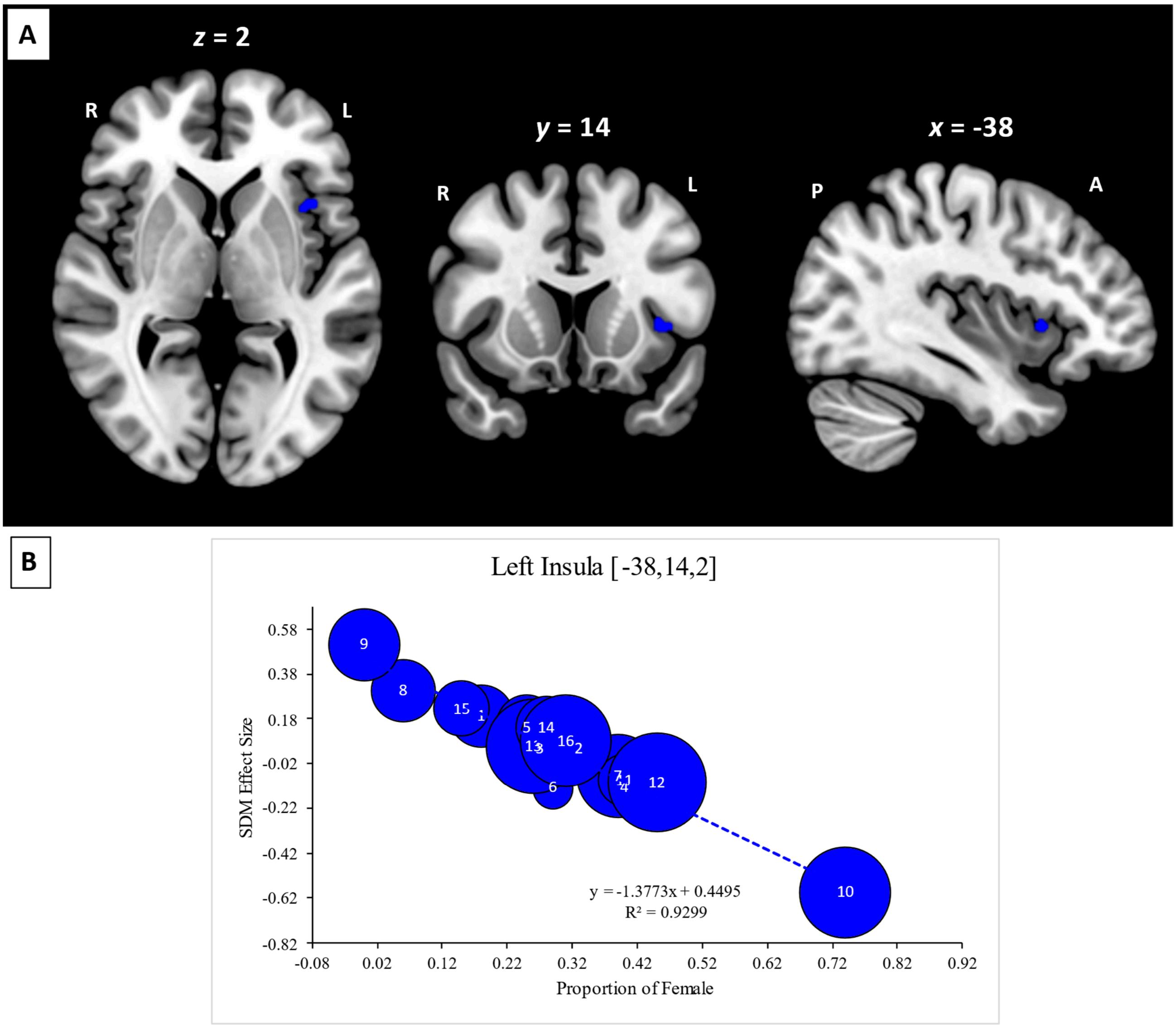

Figure S1. Caption. **(A)**. Associations between proportion of female participants in sample and BOLD response differences in the left insula (CU < TD: Insula, 19 voxels, SDM-Z = -2.90) during executive control are shown in blue All results are thresholded at $p < 0.005$. Images visualized using MRIcroGL and presented on SDM template. **(B)**. A labeled meta-regression plot for the left insula cluster (-38, 14, 2) is presented below the brain images showing a negative correlation between the effect size of BOLD response (SDM-estimate) and proportion of female

participants in each executive control fMRI study. Effect sizes (SDM-estimates) used to create the meta-regression plot were extracted from the peak of maximum slope significance. The meta-regression SDM-estimate value is derived from the proportion of studies that reported BOLD signal changes near the voxel so it is expected that some values are at 0 or near +/- 1. Each included study is represented as a numbered dot, with the dot size reflecting relative total sample size of each specific study in comparison to the average total sample size of all studies included in the regression. Study key: 1 = Padula et al., 2007; 2 = Schweinsburg et al., 2005; 3 = Schweinsburg et al., 2008; 4 = Smith et al., 2010; 5 = Tapert et al., 2007; 6 = Abdullaev et al., 2010; 7 = Cyr et al., 2019; 8 = Behan et al., 2014 ; 9 = Jager et al., 2010; 10 = Kroon et al., 2021; 11 = Hatchard et al., 2014; 12 = Tervo-Clemmens et al., 2018; 13 = Thayer et al., 2015; 14 = Jacobsen et al., 2007; 15 = Schweinsburg et al., 2010; 16 = Schweinsburg et al., 2011. Abbreviations: A= anterior; P= posterior; L= left; R= right; CU= Cannabis Use; TD= Typically Developing

Table S5. Results of Jack-knife Reliability Analyses of the Executive Control Meta-analysis

**Studies**	**rmPFC (4, 60, -4)**
Padula et al., 2007	Yes* (2, 58, -6) and (4,54,0) $^{\Delta}$
Schweinsburg et al., 2005	No
Schweinsburg et al., 2008	Yes* (6, 60, -4)
Schweinsburg et al., 2010	No$^{\pm\,\Delta}$ (44, -20, 14)
Schweinsburg et al., 2011	No$^{\pm\,\Delta}$ (44, -20, 14)
Smith et al., 2010	Yes* (6, 60, -4)
Tapert et al. 2007	Yes* (6, 60, -4)
Jager et al., 2010	Yes* (6, 60, -4) and (2,52,-2)$^{\Delta}$
Jacobsen et al., 2007	No
Kroon et al., 2021	Yes*$^{\Delta}$ (2, 54, -2) and (0,58,-8)
Tervo-Clemmens et al., 2018	Yes*$^{\Delta}$ (0, 58, -8)
Cyr et al., 2019	No
Hatchard et al., 2014	No$^{\pm}$ (52, -16, 44)
Abdullaev et al.	No
Behan et al., 2014	Yes*$^{\Delta}$ (6, 60, -4) and (2,52,-2)
Thayer et al., 2015	Yes* (4, 58, -4) and (46,-18,12)

Note: rmPFC= rostral medial prefrontal cortical cluster that showed increased BOLD response in CU compared to TD youth during tasks of executive function/cognitive control. Yes= denotes that BOLD signal differences between CU vs. TD youth in the rmPFC cluster remained significant following exclusion of this study/dataset as part of the jackknife sensitivity analysis; No= denotes that CU vs. TD BOLD signal differences in the rmPFC cluster were no longer significant when the study/dataset is removed; * = denotes that the rmPFC activation differences in this region remained significant in meta-analyses when this dataset/study was removed but the peak was located at slightly different coordinates. Δ = denotes that the brain region activation difference between CU and TD youth was significant but was small in volume (< 8 voxels). ± = denotes studies for which another significant activation foci that differentiated CU and TD youth was identified (in addition to the rmPFC cluster) during reliability testing. Specifically, when the meta-analysis was rerun excluding each of the four studies demarcated with $^{\pm}$, a significant activation foci/cluster localized to right supramarginal gyrus (SMG) and primary somatosensory cortex was identified, that showed increased BOLD response in CU compared to TD youth.

Table S6. Results of Jack-knife Reliability Analyses of the Social Cognition/Emotion Processing Meta-analysis

**Studies**	**dmPFC/dACC (2, 50, 22)**
Gilman et al., 2016a	Yes
Gilman et al., 2016b	Yes$^{\pm}$ and (42, 10, -14)
Gilman et al., 2016c	Yes$^{\pm}$ and (42, 10, -14)
Blair et al., 2021	Yes* (2, 50, 24)
Heitzeg et al., 2015	No$^{\pm}$ and (42, 10, -14)
Leiker et al., 2019	No
Blair et al., 2019	Yes* (2, 48, 20)
Aloi et al., 2018	Yes* (2, 48, 20)
Zimmerman et al., 2017	Yes$^{\pm}$ and (42, 10, -14)

Note: dmPFC/dACC= cluster combining dorsal medial prefrontal cortex (dmPFC) and dorsal anterior cingulate cortex (dACC) regions that showed increased BOLD response in CU compared to TD youth during social cognition and emotion processing tasks. Yes= denotes that BOLD signal differences between CU vs. TD youth for this brain region (dmPFC) remain significant following exclusion of this study/dataset as part of the jackknife sensitivity analysis; No= denotes that CU vs. TD BOLD signal differences for this brain region were no longer significant when the study/dataset is removed; * = denotes that the brain region activation difference finding remained significant in meta-analyses when this dataset/study was removed but the peak of the foci was located at slightly different coordinates. ± = denotes studies for which another significant activation foci was identified (in addition to the dmPFC/dACC cluster) during reliability testing. Specifically, jackknife analysis excluding these four studies showed activation differences in a small cluster (<10 voxels) localized to the right insula (42, 10, -14) that showed decreased BOLD response in CU compared to TD youth.

Figure S2. Funnel Plots for Primary Meta-analysis Related to Executive Function/Cognitive Control (A) and Social Cognition Emotion Processing (B) Domains.

A.

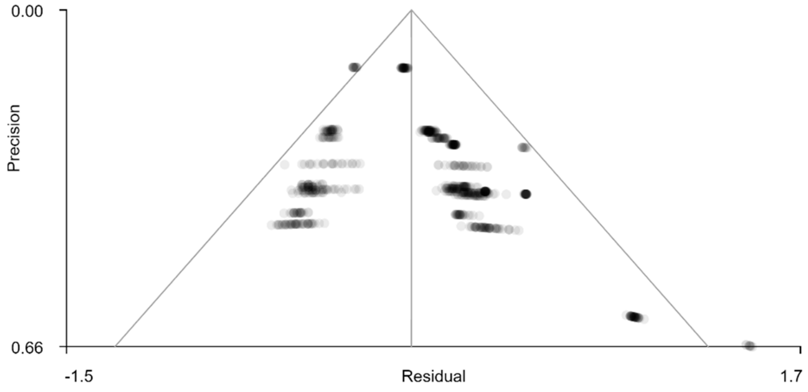

B.

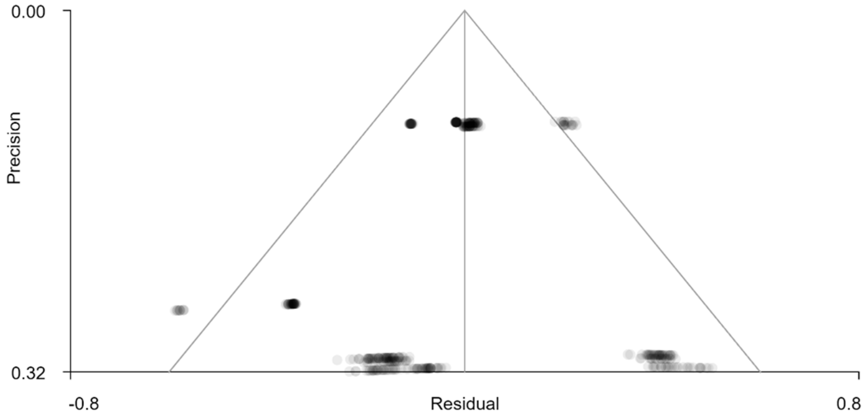

Figure S2. Caption: Funnel Plots for Primary Meta-analysis of Executive Function/Cognitive Control Domain **(A)** and Social Cognition/Emotion Processing Domain **(B)**. Funnel plots were created using SDM software and plotted the effect estimate (standardized BOLD signal difference between CU and TD participants) on the X-axis and the variance on the Y-axis for each study included in the primary meta-analyses focused on EF/CC and SC/EM domains. Results of these funnel plots show symmetric distribution of studies suggesting low evidence for bias in our two primary meta-analytic results. Using SDM's Metabias calculation tool, the Risk of Bias for the EF/CC domain meta-analysis is: Bias Test = 0.86, z: 1.03, df: 14, *p=0.301* and the Risk of Bias for the SC/EM domain meta-analysis is: Bias Test = -0.19, z: -0.20, df: 7*, p=0.838*.

Table S7. Abstinence-based subgroup Meta-analyses of fMRI studies comparing CU and TD youth

			MNI coordinates				
**Cluster #, Label**	**BA**	**Voxels**	*x*	*y*	*z*	**SDM-Z**	**P-value**
**Ad-lib use to ≥ 12-hours abstinent**							
CU > TD youth							
Right caudate<br>Right thalamus<br>Right anterior thalamic projections<br>Corpus callosum	25	120	16	16	4	3.30	*p= 0.0005*
			16	16	4		
			10	14	2		
			8	6	2		
			0	2	2		
CU < TD youth							
None							
**≥ 24-hours abstinent**							
CU > TD youth							
Right insula<br>Right IFG, orbital part	47, 38	74	42	18	-6	3.02	*p=0.0013*
			42	18	-10		
			46	18	-10		
CU < TD youth							
None							
**≥ 48-hours to ≥72-hours abstinent**							
CU > TD youth							
Right insula<br>Right IFG, triangular part	48, 47, 45	29	36	28	6	3.23	*p=0.0006*
			40	32	2		
CU < TD youth							
None							
**≥21 days abstinent**							
CU > TD youth							
None							
CU < TD youth							
Cluster #1<br>Right dmPFC<br>Left dmPFC<br>Right dACC<br>Left dACC	10, 32, 9	437	4	52	28	-4.70	*p=0.000001*
			-4	50	28		
Cluster #2<br>Right precentral gyrus<br>Right postcentral gyrus	3, 4	26	48	-14	44	-3.23	*p=0.0006*
			52	-16	40		

NOTE: SDM meta-analyses were carried out in SDM-PSI.v.6.21 on subgroups of fMRI studies comparing CU and TD youth stratified based upon the length of abstinence required at the time of the scan (Ad-lib cannabis use to ≥12-hours abstinent CU subgroup [10 studies], ≥24-hours abstinent CU subgroup [11 studies], ≥48-hours to ≥72-hours abstinent CU subgroup [7 studies], and ≥ 21 days of abstinence or longer CU subgroup [15 studies]). Statistical analysis threshold set at P-value <0.005. Coordinates shown are MNI. Abbreviations: BA= Broadman's area; dmPFC= dorsal medial prefrontal cortex; dACC= dorsal Anterior cingulate cortex; IFG= inferior frontal gyrus; CU= cannabis using youth; TD= typically developing control youth

Figure S3. Meta-analysis Results showing BOLD response differences in CU compared to TD youth for different abstinence subgroups.

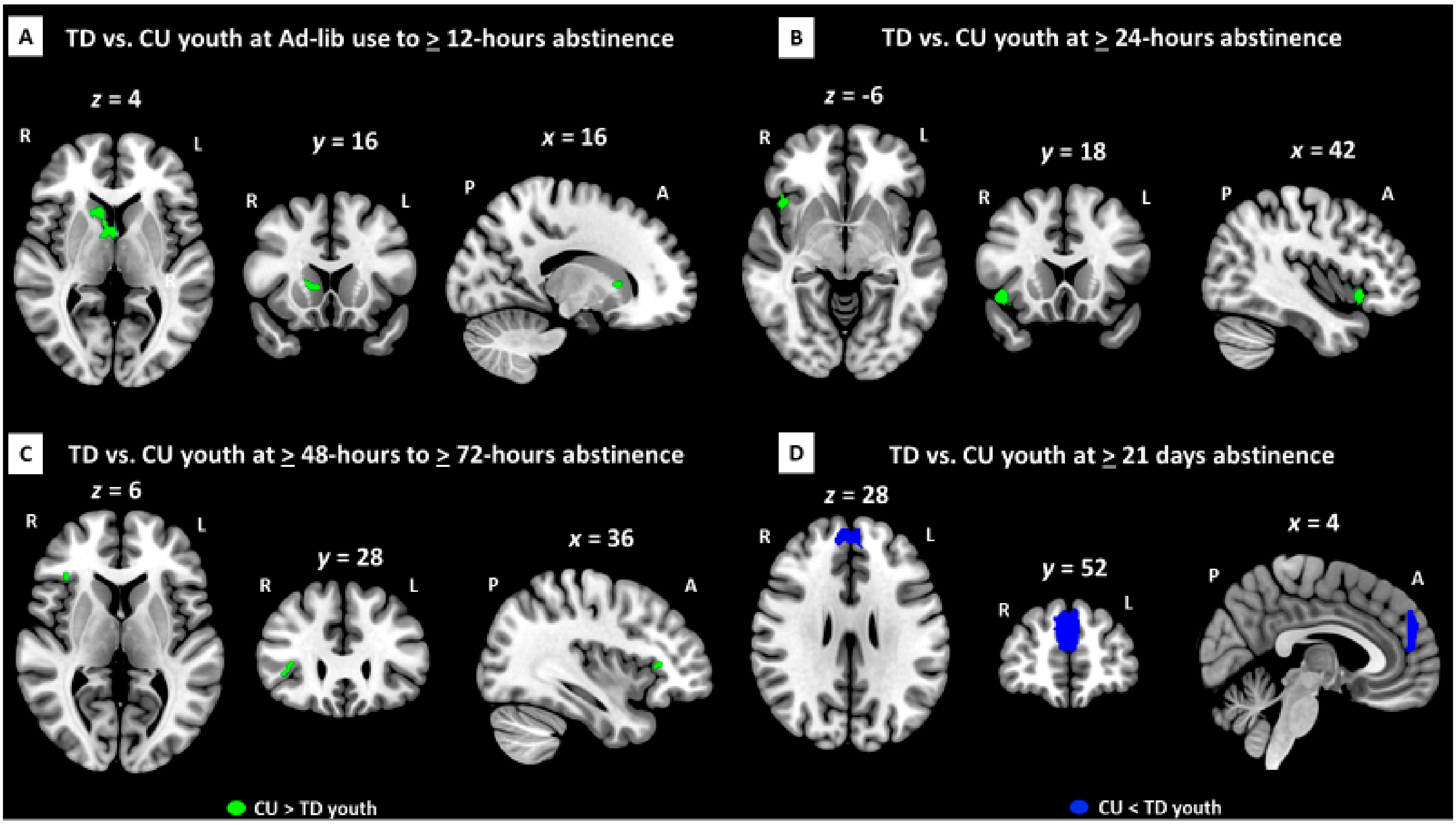

Figure S3. Caption: **(A)** Meta-analytic Result comparing TD youth to CU youth at Ad-lib use to > 12-hours abstinence. At ad-lib use to 12-hour abstinence, an increase in activation in CU youth compared to TD youth can be seen in green in the right caudate extending to the anterior thalamic projections, thalamus, and corpus callosum (peak cluster of 120 voxels; MNI coordinates: x=16, y=16, z=4). **(B)** Meta-analytic Result comparing TD youth to

CU youth at > 24-hours abstinence. At 24-hours abstinence, an increase in activation in CU youth compared to TD youth can be seen in green in the right insula extending anteriorly into the right IFG (peak cluster of 74 voxels; MNI coordinates: x=42, y=18, z=-6). **(C)** Meta-analytic Result comparing TD youth to CU youth at > 48-hours to > 72-hours abstinence. Similar to at 24-hours, at 48-to-72-hours abstinence, an increase in activation in CU youth compared to TD youth can be seen in green in the right insula extending anteriorly into the right IFG (peak cluster of 29 voxels; MNI coordinates: x=36, y=28, z=6). **(D)** Meta-analytic Results comparing TD youth to CU youth at > 21-days abstinence. At 21 days or longer abstinence, a decrease in activation in CU compared to TD youth can be seen in blue in a large bilateral cluster localized to the right/left dorsal mPFC and right/left dorsal ACC (peak cluster of 437 voxels; MNI coordinates: x=4, y=52, z=28). Activation differences in a small cluster localized to the right precentral and postcentral gyri (peak cluster of 26 voxels; MNI coordinates: x=48, y=-14, z=44) were also found in CU at ≥ 21-days abstinence but are not visible on these images. All results are thresholded at $p < 0.005$ (cluster size > 10 voxels). Green is used to identify activation foci where CU > TD youth. Blue is used to identify activation foci where CU < TD youth. Images visualized using MRIcroGL and presented on SDM template. Abbreviations: BOLD = blood-oxygen-level-dependent; CU = cannabis using; TD = typically developing; MNI = Montreal Neurologic Institute coordinates; mPFC = medial prefrontal cortex; ACC= anterior cingulate cortex; IFG = inferior frontal gyrus.

Figure S4. Meta-analysis Results showing BOLD response differences between adolescents with cannabis use disorders and matched non-using typically developing adolescent controls.

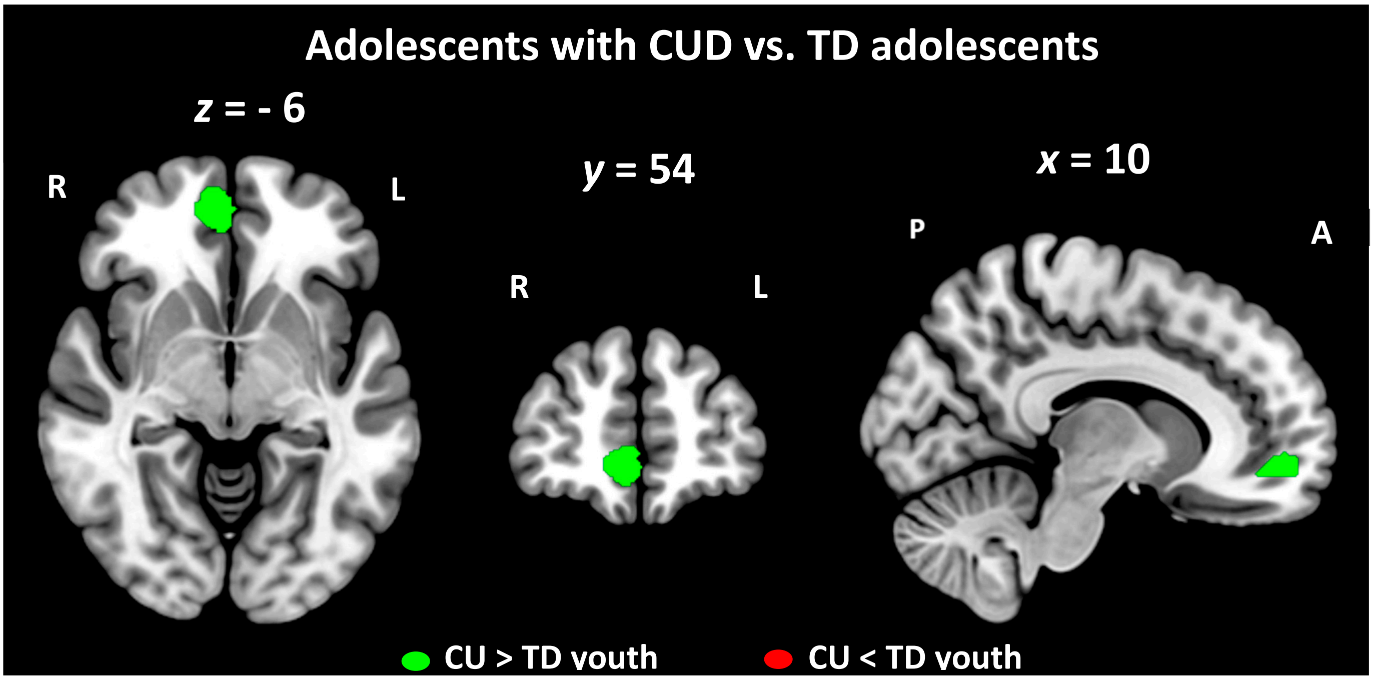

Figure S4. Caption: Subgroup Meta-analysis results comparing adolescents with CUD to TD adolescents across all domains/paradigms. An increase in activation in adolescents with CUD versus TD adolescents across all domains/paradigms in the rostral, ventral, and dorsal mPFC extending to the dACC centered in the right rmPFC (peak cluster of 258 voxels; MNI coordinates: x=2, y=50, z=-4) is shown in green. A second cluster showing the increase in brain activation in the inferior parietal lobule (IPL) in adolescents with CUD compared to TD youth was also found but is not visualized in this figure. All results are thresholded at $p < 0.005$. Images visualized using MRIcroGL and presented on SDM template. Abbreviations: BOLD = blood-oxygen-level-dependent; CU = cannabis using; CUD= cannabis use disorder; dACC= dorsal anterior cingulate cortex; TD = typically developing; MNI = Montreal Neurologic Institute coordinates; mPFC = medial prefrontal cortex; rmPFC= rostral mPFC; R= right; L= left; A= anterior; P= posterior
